# A Dual Barcoding Approach to Bacterial Strain Nomenclature: Genomic Taxonomy of *Klebsiella pneumoniae* Strains

**DOI:** 10.1093/molbev/msac135

**Published:** 2022-06-14

**Authors:** Melanie Hennart, Julien Guglielmini, Sébastien Bridel, Martin C J Maiden, Keith A. Jolley, Alexis Criscuolo, Sylvain Brisse

**Affiliations:** Institut Pasteur, Université Paris Cité, Biodiversity and Epidemiology of Bacterial Pathogens, Paris, France; Sorbonne Université, Collège Doctoral, Paris, France; Institut Pasteur, Université Paris Cité, Bioinformatics and Biostatistics Hub, Paris, France; Institut Pasteur, Université Paris Cité, Biodiversity and Epidemiology of Bacterial Pathogens, Paris, France; Department of Zoology, University of Oxford, Oxford, United Kingdom; Department of Zoology, University of Oxford, Oxford, United Kingdom; Institut Pasteur, Université Paris Cité, Bioinformatics and Biostatistics Hub, Paris, France; Institut Pasteur, Université Paris Cité, Biodiversity and Epidemiology of Bacterial Pathogens, Paris, France

**Keywords:** genomic classification, strain nomenclature, microevolution, pathogen tracking, genomic library, international harmonization

## Abstract

Sublineages (SLs) within microbial species can differ widely in their ecology and pathogenicity, and their precise definition is important in basic research and for industrial or public health applications. Widely accepted strategies to define SLs are currently missing, which confuses communication in population biology and epidemiological surveillance. Here, we propose a broadly applicable genomic classification and nomenclature approach for bacterial strains, using the prominent public health threat *Klebsiella pneumoniae* as a model. Based on a 629-gene core genome multilocus sequence typing (cgMLST) scheme, we devised a dual barcoding system that combines multilevel single linkage (MLSL) clustering and life identification numbers (LINs). Phylogenetic and clustering analyses of >7,000 genome sequences captured population structure discontinuities, which were used to guide the definition of 10 infraspecific genetic dissimilarity thresholds. The widely used 7-gene multilocus sequence typing (MLST) nomenclature was mapped onto MLSL SLs (threshold: 190 allelic mismatches) and clonal group (threshold: 43) identifiers for backwards nomenclature compatibility. The taxonomy is publicly accessible through a community-curated platform (https://bigsdb.pasteur.fr/klebsiella), which also enables external users’ genomic sequences identification. The proposed strain taxonomy combines two phylogenetically informative barcode systems that provide full stability (LIN codes) and nomenclatural continuity with previous nomenclature (MLSL). This species-specific dual barcoding strategy for the genomic taxonomy of microbial strains is broadly applicable and should contribute to unify global and cross-sector collaborative knowledge on the emergence and microevolution of bacterial pathogens.

## Introduction

Taxonomy is a foundation of biology that entails the classification, nomenclature, and identification of biological objects (Cowan 1965). Although the Linnaean system is organized into taxonomic ranks down to the level of species (Sneath 1992), sublineages (SLs) within microbial species can diversify as independently evolving lineages that persist over long periods of time (Selander and Levin 1980), and the broad microbial species definition and horizontal gene transfer (HGT) of accessory genes underlie extensive strain heterogeneity of phenotypes with ecological, medical, or industrial relevance (Hacker and Kaper 2000; Lan and Reeves 2001; Feil 2004; Konstantinidis and Tiedje 2005). Nevertheless, strain-level diversity is overlooked by the current prokaryotic taxonomy.

Most attempts to develop and maintain microbial strain taxonomies aimed at facilitating epidemiological surveillance and outbreak detection (Maiden et al. 1998, 2013; van Belkum et al. 2007). Although local epidemiology can rely on vernacular type designations, the benefits of unified nomenclatures of SLs for large-scale epidemiology and population biology were recognized early (Struelens et al. 1998). By far the most successful taxonomic system of microbial strains is the multilocus sequence typing (MLST) approach (Maiden et al. 1998; Achtman et al. 2012). This highly reproducible and portable nomenclature system has been extensively used for studies of population biology and public health surveillance of bacterial pathogens (Jolley et al. 2018). Core genome MLST (cgMLST) extends the advantages of the MLST approach at the genomic scale (Jolley and Maiden 2010; Maiden et al. 2013) and provides strain discrimination at much finer scales.

Strain classification, based either on cgMLST or on nucleotide polymorphisms, can be achieved by using several clustering thresholds simultaneously, leading to a succession of group identifiers (“barcodes”) that provide relatedness information at increasing levels of phylogenetic depth (Maiden et al. 2013; Moura et al. 2016). This approach was recently formalized as hierarchical clustering (HierCC, based on cgMLST) (Zhou et al. 2021) and as the “single nucleotide polymorphism (SNP) address” (Dallman et al. 2018), based on single linkage classifications; here, we generically refer to these approaches as MultiLevel Single Linkage (MLSL). Unfortunately, the single linkage clustering may result in the fusion of preexisting groups as additional genomes are introduced, due to the possibility of new genomes being less distant than the threshold, from two distinct groups. This approach thus suffers from instability, which led HierCC inventors to instead use ad hoc group attribution rules after an initial single linkage classification (Zhou et al. 2021).

An alternative approach, the life identification number (LIN) encoding, was proposed by Vinatzer and colleagues (Vinatzer et al. 2017; Tian et al. 2020): a multiposition numerical code is assigned to each genome based on its similarity with the closest genome already encoded. An attractive property of this procedure is that LIN codes are definitive, that is, not affected by subsequent additions of genomes, as they are attributed to individual genomic sequences rather than to groups. However, in the current implementation of LIN codes, the similarity between genomes is estimated using average nucleotide identity (ANI), which may be imprecise for nearly identical strains.

Here, we present a strain classification, naming, and identification system for bacterial strains, which is based on cgMLST and combines the MLSL and LIN code approaches. We took as a model the *Klebsiella pneumoniae* species complex, a genetically and ecologically highly diverse bacterial group that causes a wide range of infections in humans and animals (Brisse et al. 2006; Wyres, Lam, et al. 2020). Given its extensive diversity and fast evolutionary dynamics, *K. pneumoniae* is a challenging model for the development of a genomic taxonomy of strains. Moreover, the rapid emergence and global dissemination of multidrug resistance in *K. pneumoniae,* sometimes combined with high virulence (Bialek-Davenet et al. 2014; Wyres, Nguyen, et al. 2020), have created a pressing need for an efficient *K. pneumoniae* strain definition and tracking system.

## Results

### Genome-Based Phylogenetic Structure of the *K. pneumoniae* Species Complex

**Fig. 1.**
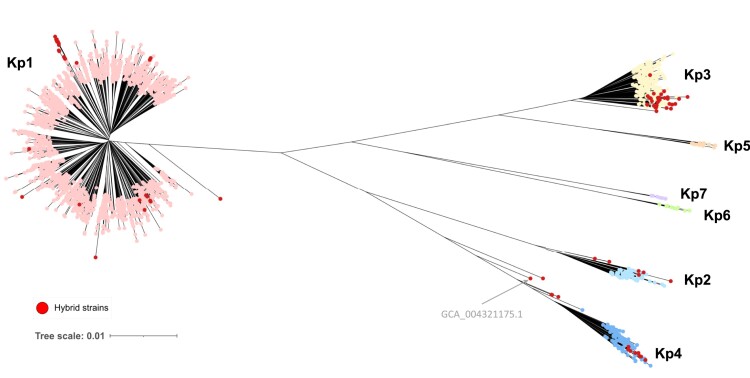
Genome-based phylogenetic tree of the *Klebsiella pneumoniae* species complex. The whole-genome distance-based tree was inferred using JolyTree. JolyTree uses *mash* to decompose each genome into a sketch of k-mers and to quickly estimate the *p*-distance between each pair of genomes; after transforming every *p*-distance into a pairwise evolutionary distance, a phylogenetic tree is inferred using FastME. The seven phylogroups are indicated. Red dots correspond to strains defined as interphylogroup hybrids. Scale bar, 0.01 nucleotide substitutions per site.

**Fig. 2.**
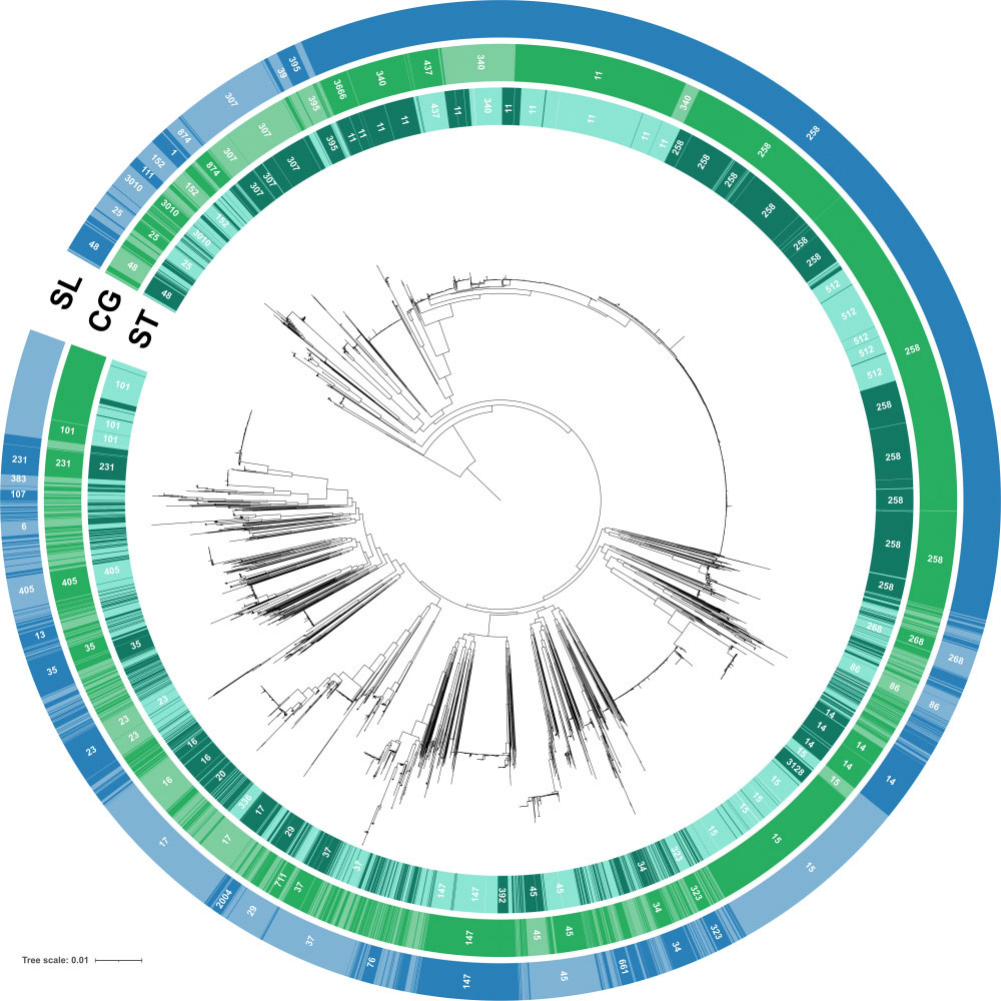
Phylogenetic structure within phylogroup Kp1 (*K. pneumoniae sensu stricto*). The circular tree was obtained using IQ-TREE based on the concatenation of the genes of the scgMLSTv2 scheme; 1,600 isolates are included (see Materials and Methods). Labels on the external first circle represent 7-gene MLST ST identifiers (each alternation corresponds to a different ST and only ST with more than 20 strains are labeled). The second and third circles (light green and blue, respectively) show the alternation of CGs and SLs, respectively, labeling only groups with more than 20 isolates. Full correspondence between ST, SL, and CG identifiers is given in the supplementary appendix, Supplementary Material online.

The deep phylogenetic structure of the *K. pneumoniae* (Kp) species complex (KpSC) (fig. 1) reflects the previously recognized seven major phylogroups, Kp1–Kp7 (Brisse and Verhoef 2001; Fevre et al. 2005; Blin et al. 2017; Long et al. 2017; Rodrigues et al. 2019; Wyres, Lam, et al. 2020). The most represented phylogroup (91.7%; *n* = 6,476) is Kp1, that is, *K. pneumoniae sensu stricto* (table 1), and its phylogenetic structure (fig. 2) revealed a multitude of SLs (note that below, we define sublineages and clonal groups [CGs] is a stricter sense in “Definition of classification thresholds for phylogroups, sublineages, and clonal groups”). There were multiple closely related isolates within some SLs, most prominently within an SL comprising genomes with 7-gene MLST identifiers ST258, ST11, ST512 (fig. 2), which represented more than one-third (33.4%) of the Kp1 data set. The abundance of this SL (and a few others, such as ST23) reflected the clinical microbiology focus on multidrug-resistant or hypervirulent isolates (Bowers et al. 2015; Struve et al. 2015; Lam et al. 2018; Wyres, Nguyen, et al. 2020). The phylogenetic structure within other *K. pneumoniae* phylogroups also revealed a multitude of distinct SLs but no predominant ones, and medically important lineages in these phylogroups are yet to be recognized.

**Table 1.** Genome Dataset Phylogroup Breakdown, Quality Assessment and Diversity.

Taxonomic designation	Phylogroup	Initial no. genomes	No. QC-filtered	No. after applying filters	No. of hybrids	No. of nonhybrid genomes	No. of called alleles (mean, std)	No. of sequence types (ST)
*K. pneumoniae* subsp. *pneumoniae*	Kp1	6,737	218 (3.2%)	6,519 (90.6%)	43 (0.7%)	6,476 (91.7%)	624.6 (3.6)	705
*K. quasipneumoniae* subsp. *quasipneumoniae*	Kp2	115	1 (0.9%)	114 (1.6%)	8 (7.0%)	106 (1.5%)	604.0 (2.5)	49
*K. variicola* subsp. *variicola*	Kp3	309	8 (2.6%)	301 (4.2%)	37 (12.3%)	264 (3.7%)	615.4 (3.3)	149
*K. quasipneumoniae* subsp. *similipneumoniae*	Kp4	230	6 (2.6%)	224 (3.1%)	50 (22.3%)	174 (2.5%)	607.4 (1.9)	64
*K. variicola* subsp. *tropica*	Kp5	19	0 (0.0%)	19 (0.3%)	0 (0.0%)	19 (0.3%)	611.7 (1.8)	13
*K. quasivariicola*	Kp6	13	2 (15.4%)	11 (0.2%)	0 (0.0%)	11 (0.2%)	602.9 (1.8)	8
*K. africana*	Kp7	10	0 (0.0%)	10 (0.1%)	0 (0.0%)	10 (0.1%)	606.3 (1.6)	4
Total		7,433	235	7,198	138	7,060	mean 610.3 (2.36)	992

*Klebsiella pneumoniae* strains can recombine large sections of their chromosome (Chen et al. 2014; Wyres et al. 2015). Large recombination events were detected in 1.9% (138/7,198) genomes (based on their cgMLST profiles) and involved the phylogroups Kp1, Kp2, and Kp4 (supplementary appendix: Detection of hybrids, fig. S1, and tables S1 and S2, Supplementary Material online). The phylogenetic impact of large-scale recombination is illustrated in figure 1, with “hybrids” occurring on misleadingly long branches.

### cgMLST Analysis of the *K. pneumoniae* Species Complex

A previously defined cgMLST (named scgMLST, with 634 loci) scheme (Bialek-Davenet et al. 2014) was updated (supplementary table S3, Supplementary Material online) and defined as scgMLSTv2 (with 629 loci, as five of the original ones were removed; see Materials and Methods). cgMLST allelic profiles were then determined for 7,433 genomic sequences (including 45 reference sequences; supplementary fig. S2, Supplementary Material online). The mean number of missing alleles per profile was 8 (1.2%; standard deviation: 25; 4.0%), and most (7,198; 96.8%) isolates had a cgMLST profile with fewer than 30 (4.8%) missing alleles. Missing allele proportions did not vary significantly among phylogroups (table 1). The transcription-repair coupling factor *mfd* gene was atypical, with 778 alleles and an average allele size of 3,447 nucleotides (nt); for the other loci, the number of distinct alleles varied from 8 to 626 (median: 243) and was strongly associated with locus size (range: 123–2,826 nt; median: 758 nt; supplementary fig. S3, Supplementary Material online). Locus-by-locus recombination analyses detected evidence of intra-gene recombination (PHI test; 5% *P*-value significance) in half of the loci (318/629; 50.6%) and these exhibited more alleles than nonrecombining ones (supplementary table S3 and fig. S3, Supplementary Material online).

**Fig. 3.**
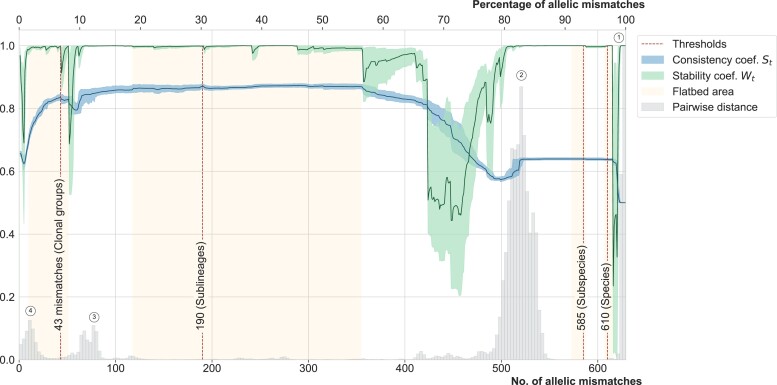
Distribution of pairwise cgMLST distances, clustering properties, and phylogenetic congruence.Values are plotted for the 7,060 genomesdata set. Threshold values (*t*) are shown on the *X*-axis, corresponding to allelic profilemismatch values up to 629 (or 100%). Grey histograms: distribution of pairwise allelic mismatches. The circles correspond to thedifferent modes of distribution. The curves represent the consistency coefficient *S_t_* (silhouette, blue) and stability coefficient *W_t_* (green),respectively, obtained with each threshold *t*; the corresponding scale is on the left *Y*-axis. To identify the two curves without referenceto their colour, note that the St curve starts (at *X* = 0) approximately at 0.65 and the Wt curve starts at approx. 0.95. The dotted verticalred lines at *t* = 43/629, 190/629, 585/629, and 610/629 represent the thresholds up to which pairs of genomes belongto the same CGs,SLs, phylogroups, and species, respectively.

The distribution of pairwise allelic mismatch proportions among nonhybrid cgMLST allelic profiles was discontinuous (fig. 3), with four major modes centered around values 99.7% (627 mismatches; i.e., 0.3% similarity), 82.7% (520 mismatches; 17.3%), 12.4% (79 mismatches; 87.6%), and 2.0% (13 mismatches; 98%). ANI values (supplementary fig. S4, Supplementary Material online) varied from 92.8% to 100%, with two first modes at 93.5% and 95.5%, composed of interphylogroup strain comparisons. The corresponding genome pairs typically had only ≈2% cgMLST similarity. In turn, whereas the range of ANI values was only 98–100% for intraspecies pairs, their cgMLST similarities occupied the much broader 5–100% range.

The 627-mismatch mode corresponded mostly to pairs of strains belonging to distinct species of the KpSC (fig. 3; supplementary fig. S4, Supplementary Material online), while a minor peak centered on 591 mismatches (supplementary fig. S5, Supplementary Material online) corresponded to comparisons between subspecies of *Klebsiella quasipneumoniae* and *Klebsiella variicola* (Kp2 and Kp4, and Kp3 and Kp5, respectively; supplementary fig. S4, Supplementary Material online). Whereas the 520-mismatch mode corresponded to inter-ST comparisons in 99.9% cases, the 13-mismatch mode was largely dominated by comparisons of cgMLST profiles with the same ST (68.2%; pairs of genomes within 402 distinct STs) or of single-locus variants (SLV; 30.8%). Finally, the 79-mismatch mode comprised a large proportion (48.0%) of ST258–ST11 comparisons and other comparisons of atypically closely related STs (supplementary fig. S5, Supplementary Material online).

### Definition of Classification Thresholds for Phylogroups, SLs, and Clonal Groups

To determine optimal allelic mismatch thresholds that would reflect the KpSC population structure, the consistency and stability properties of single linkage clustering groups were assessed for every threshold value *t* from 1 to 629 allelic mismatches. The consistency (silhouette) coefficient *S_t_* had a plateau of optimal values in the range corresponding to 118/629 (18.8%) to 355/629 (56.4%) allelic mismatches (fig. 3, blue curve). Analysis of the robustness to subsampling (*W_t_*; based on an adjusted Wallace coefficient; fig. 3, green curve) identified several ranges of allelic mismatch threshold values that were associated with maximal stability.

The above analyses led us to propose four deep classification levels. The two first thresholds, 610 and 585 allelic mismatches, enable species and subspecies separations, respectively. We next defined a threshold of 190 allelic mismatches, corresponding to the optimal combination of consistency and stability coefficients *S_t_* and *W_t_*. The single linkage clustering based on this threshold created 705 groups, which we here define as “SLs”. By design, this threshold separated into distinct groups, the pairs of cgMLST profiles corresponding to the major mode (at 520 mismatches), that is, the majority of genomes that have distinct STs within phylogroups. Finally, a threshold of 43 allelic mismatches was defined to separate genome pairs of the 79-mismatch mode. This value corresponded to the local optima of both *S_t_* and *W_t_* coefficients. Interestingly, this last threshold value was also located in the optimal range of compatibility with the classical 7-gene ST definitions (Rand index *R_t_* ≥ 0.70 was observed for 10 ≤ *t* ≤ 51). The use of this threshold resulted in 1,147 groups, which we propose to define as “CGs”.

**Fig. 4.**
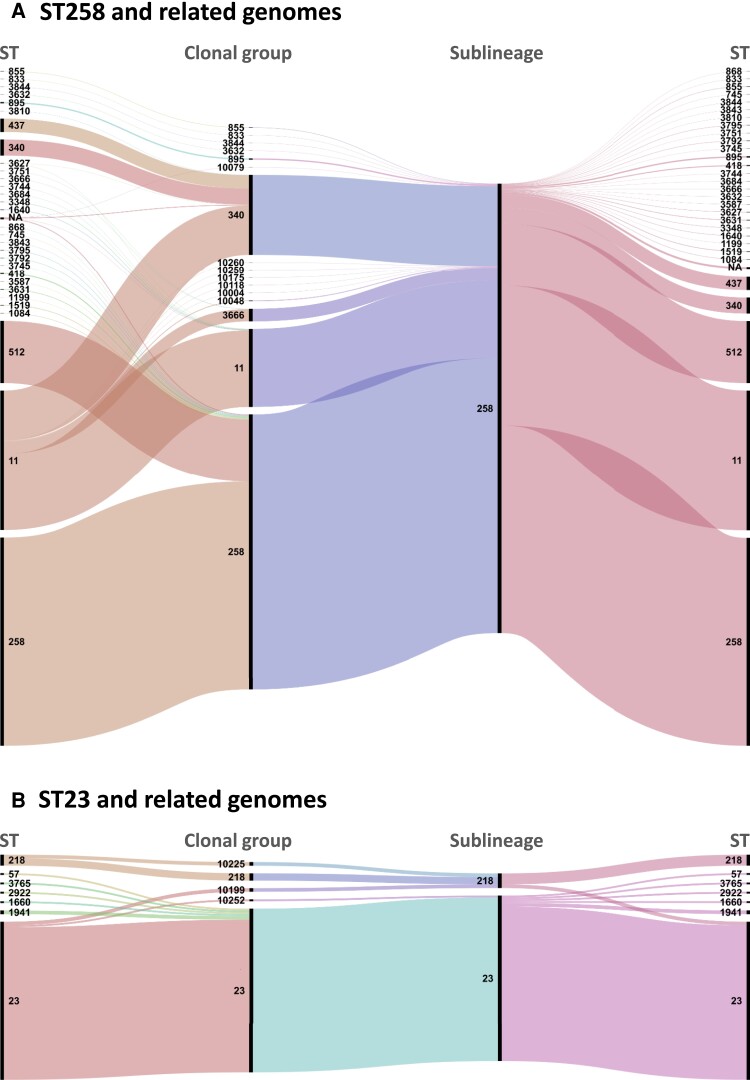
Concordance of SL, clonal group and 7-gene MLST classifications (Panel A: ST258 and related genomes; Panel B: ST23 and related genomes). Alluvial diagram obtained using RAWGraphs (Mauri et al. 2017) showing the correspondence between STs (7 genes identity), CGs (43 allelic mismatches threshold), and SLs (190 allelic mismatches threshold). Colors are arbitrarily attributed by the software for readability.

Overall, approximately half (547/1,147; 47.7%) of the CGs corresponded one-to-one with the SL level (supplementary table S4, Supplementary Material online): 77.6% (547/705) SLs contained a single CG, whereas 158 (22.4%) SLs comprised at least two CGs (supplementary table S5 and fig. S6, Supplementary Material online; fig. 4). Overall, CG compatibility with classical ST classification was high (i.e., *R_t_* = 0.72, whereas it was only 0.50 for SLs).

The distribution of pairwise allelic mismatch values that involved hybrid genomes showed an additional peak around 39 shared alleles (i.e., 590 allelic mismatches; supplementary fig. S7, Supplementary Material online). Therefore, these interphylogroup hybrids were placed into distinct partitions at the 585-mismatch level. However, as some of these hybrid genomes diverged by fewer than 585 allelic mismatches from two distinct phylogroups at the same time, they would cause the fusion of phylogroup partitions upon single linkage clustering. To highlight the impact of this phenomenon, hybrids were first filtered out and next incorporated in a second single linkage clustering step (supplementary appendix and fig. S8, Supplementary Material online).

### Phylogenetic Compatibility of SLs and Clonal Groups

To estimate the congruence of classification groups with phylogenetic relationships among genomic sequences, we quantified the proportion of monophyletic (single ancestor, exclusive group), paraphyletic (single ancestor, nonexclusive group), and polyphyletic (two or more distinct ancestors) groups. Regarding 7-gene MLST, 6,985 (98.9%) genomes had a defined ST, that is, an allele was called for each of the seven genes. Of the 992 distinct STs, 396 were nonsingleton STs (i.e., comprised at least two isolates). Of these, 286 (72.2%) were monophyletic, 9 were paraphyletic (2.3%), and 101 (25.5%) were polyphyletic. The monophyletic STs comprised only 22% of all genomes in nonsingleton STs.

Regarding cgMLST-based classification, there were 5 and 7 partitions at 610 and 585 allelic mismatch levels, respectively, and 100% of these were monophyletic. Among the 705 distinct SLs, 317 (45.0%) were nonsingleton, and most (310; 97.8%) of these were monophyletic (see fig. 2 for Kp1); only three (0.9%) were paraphyletic, and four (1.3%) were polyphyletic (supplementary table S4, Supplementary Material online). The monophyletic SLs comprised a large majority (5,961/6,672; 89.3%) of genomes in nonsingleton STs.

Finally, 396 out of 1,147 (34.5%) CGs were nonsingleton; most (362; 91.4%) were monophyletic (fig. 2), whereas 8 (2.0%) were paraphyletic, and 26 (6.6%) were polyphyletic (supplementary table S5, Supplementary Material online). Monophyletic CGs comprised nearly half (3,030; 48.0%) of the genomes in the nonsingleton CGs, whereas 3,224 (51.1%) were in polyphyletic groups, mostly in CG258, CG340, and CG15.

### Definition of Shallow-Level Classification Thresholds for Klebsiella Epidemiology

Although the scgMLSTv2 scheme comprises only 629 loci, or ∼10% of a typical *K. pneumoniae* genome length (512,856 nt out of 5,248,520 in the NTUH-K2044 genome), shallow-level classifications of genomic sequences might be useful for tentative outbreak delineation and epidemiological surveillance purposes, by ruling-out outliers. To provide flexible case cluster definitions, we classified KpSC cgMLST profiles using thresholds of 0, 1, 2, 4, 7, and 10 scgMLSTv2 allelic mismatches. Together with the four higher levels, the MLSL nomenclature, therefore, comprises 10 classification levels in total. The classification groups corresponding to the 0-mismatch threshold correspond to groups of cgST profiles that only differ by missing data. We observed that profiles of isolates involved in previously reported KpSC outbreaks generally differed by no or 1 mismatch, with a maximum of five allelic mismatches (supplementary tables S6 and S7, Supplementary Material online), indicating that this classification approach may be useful for genomic surveillance and outbreak identification purposes.

### Inheritance of the 7-Gene ST Identifiers into the cgMLST Classification, and Characteristics of Main SLs and CGs

To attribute SL and CG identifiers that corresponded maximally to the widely adopted 7-gene ST identifiers, we developed an inheritance algorithm to map MLST identifiers onto SL and CG partitions (see supplementary appendix: Nomenclature inheritance algorithm, Supplementary Material online). Of the 705 SLs, most (683; 96.9%) were named by inheritance and this was the case for 879 (76.6%) of the 1,047 CGs (supplementary table S4, Supplementary Material online). The resulting correspondence of cgMLST partitions with classical MLST was evident for the major groups (fig. 4; supplementary fig. S6, Supplementary Material online). For instance, the multidrug-resistant SL258 comprised isolates belonging to MLST sequence types (STs) ST258, ST11, ST512, ST340, ST437, and 25 other STs. SL258 consisted of 16 distinct CGs, of which the four most frequent were defined as CG258 (61.2%), CG340 (17.8%), CG11 (17.3%), and CG3666 (2.8%) (fig. 4). When compared with 7-gene MLST, most isolates of CG258 were ST258 (75.6%) or ST512 (22.6%), whereas CG11 mostly comprised ST11 genomes (98.0%). In turn, CG340 included a large majority of ST11 genomes (61.8%) and only 20.0% ST340 genomes, and was named CG340 rather than CG11 because CG11 was already attributed. Likewise, the majority (83/86; 96.5%) of ST23 genomes, which are associated with pyogenic liver abscess (Lam et al. 2018), were classified into SL23, which itself consisted mainly (84/90, 93.3%) of ST23 genomes (fig. 4). The well-recognized emerging multidrug-resistant KpSC populations of ST15, ST101, ST147, and ST307 each corresponded largely to a single SL and CG (supplementary fig. S6, Supplementary Material online).

The frequency of detection of virulence and antimicrobial resistance genes differed among the main SLs and CGs (fig. 6; supplementary fig. S9, Supplementary Material online). As expected (Lam et al. 2021), SL23 (median virulence score of 5) and SL86 (median score 3) were prominent “hypervirulent” SLs, and they were largely lacking resistance genes. In contrast, a majority of strains from SLs 258, 147, 101, 307, and 37, as well as a large number of other SLs, had a resistance score of 2 or more, indicative of BLSE/carbapenemases, but these had modest virulence scores (supplementary fig. S9, Supplementary Material online). SL231 genomes stood out as combining high virulence and resistance scores. In some cases, CGs within single major SLs had contrasted virulence and resistance gene contents (fig. 6).

**Fig. 5.**
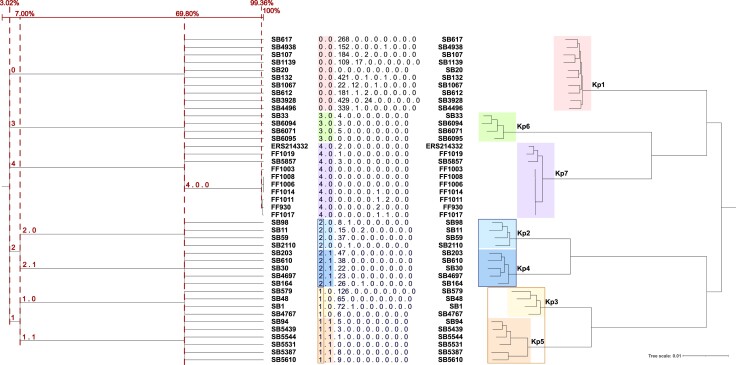
Phylogenetic relationships are reflected in cgLIN code prefixes. Left: the prefix tree generated from cgLIN codes; Right: phylogenetic relationships derived using IQ-TREE from the cgMLST gene sequences from the reference strains. The cgLIN codes are also shown. The values indicated on top of the prefix tree correspond to the cgMLST similarity percentage of the corresponding cgLIN code bin.

### Development and Implementation of a cgMLST-Based LIN Code System

Following the principle of the LIN code system, initially proposed based on the ANI similarity (Marakeby et al. 2014), we defined a cgMLST-based LIN (cgLIN) code approach. As LIN coding is performed sequentially, we first explored the impact on the resulting partitioning of cgMLST profiles, of the order in which genomes are assigned. We confirmed that the number of partitions (hence their content too) varied according to input order (supplementary fig. S10, Supplementary Material online). However, we established that the order of genomes determined by the traversal of a Minimum Spanning tree (MStree) (Prim 1957) naturally induces a LIN encoding order that is optimal, that is, most parsimonious with respect to the number of identifiers generated at each position of the code (see supplementary appendix, Supplementary Material online). Using this MStree traversal strategy, we defined cgLIN codes for the 7,060 nonhybrid genomes (as a first step), resulting in 4,889 distinct cgLIN codes.

**Fig. 6.**
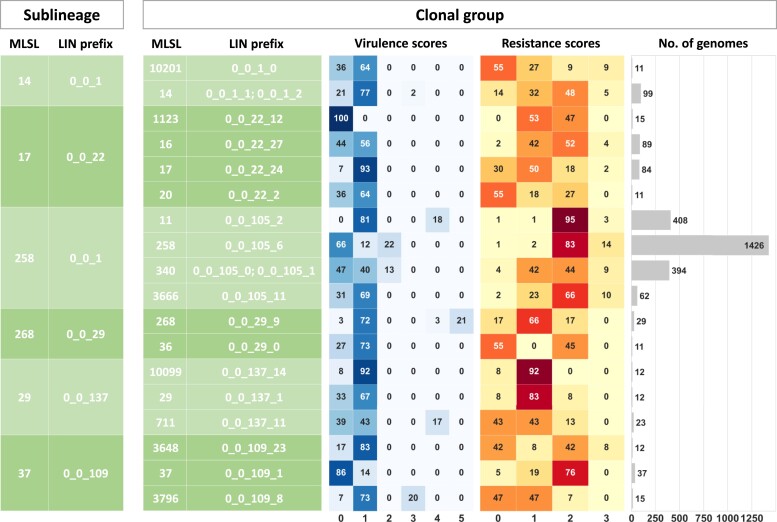
cgLIN code prefixes, and virulence and antimicrobial resistance scores of some SLs and their CGs. Left (green) four first columns: LIN prefixes of selected SLs and CGs. Right: heatmaps of virulence and resistance scores of CGs, and the number of genomes in each group. For each genome, the virulence score derived from Kleborate has a value from 0 to 5; the value in the cells corresponds to the percentage of strains in the group with that virulence score (similar to a heat map). The principle is the same for the resistance score, but it varies from 0 to 3.

Furthermore, cgLIN codes can be displayed in the form of a prefix tree (fig. 5), which largely reflects the phylogenetic relationships among genomes. In addition, cgLIN code prefixes can be used to label particular phylogenetic lineages (Vinatzer et al. 2017). For example, a single cgLIN code prefix defined each phylogroup (e.g., Kp1: prefix 0_0; Kp2: prefix 2_0; fig. 5). Likewise, a full one-to-one correspondence between prefixes and SLs was observed, and almost all (99.4%) CGs also had a unique prefix (supplementary tables S4 and S5, Supplementary Material online; fig. 6).

### Effect of Hybrid Genomes Incorporation on the MLSL and cgLIN Codes Classifications

Because interphylogroup hybrid genomes have smaller distances to their parental phylogroups than the interphylogroup distances resulting from vertical evolutionary events, their incorporation into the MLSL classification may induce the fusion of previously distinct single linkage groups. To illustrate this chaining effect, the “hybrid” genomes were included into the MLSL nomenclature in a second step, and fusions of previously existing partitions we recorded; for example, at the 610 allelic mismatch threshold, partitions 2 (Kp2 and Kp4) and 4 (Kp3 and Kp5) were merged with partition 1 (Kp1). At the 585-mismatch threshold, partitions 5 (Kp2) and 2 (Kp4) were merged with partition 1 (Kp1). At the 190-mismatch threshold, only one fusion was observed, between partitions 184 (SL113) and 465 (SL1518; supplementary fig. S11, Supplementary Material online). The partitions at other thresholds were not impacted by the addition of the hybrid genomes.

In contrast, the incorporation of hybrid genomes into the cgLIN code database left the cgLIN codes of the 7,060 previous genomes entirely unaffected; there were no merging of groups, as per the design of the system. In particular, the seven phylogroup prefixes corresponding to species and subspecies remained unaffected (supplementary fig. S11, Supplementary Material online); instead, additional prefixes were created for the hybrid genomes (supplementary tables S1 and S2, Supplementary Material online).

### Implementation of the Genomic Taxonomy in a Publicly Accessible Database

The MLSL nomenclature was incorporated into the Institut Pasteur *K. pneumoniae* MLST and whole-genome MLST databases (https://bigsdb.pasteur.fr/klebsiella) under the classification scheme functionality developed in BIGSdb version 1.21.0. In brief, the cgMLST profile of every isolate with fewer than 30 missing scgMLSTv2 alleles was assigned to a core genome sequence type (cgST), and these were next grouped into single linkage partitions for each of the 10 classification levels. For SLs and CGs, a custom classification group field (named SL or CG within the system) was additionally populated with identifiers inherited from 7-gene MLST. All cgMLST profiles and classification identifiers are publicly available.

To allow users to identify *K. pneumoniae* isolates easily, a profile matching functionality was developed, enabling them to search for cgMLST profiles related to a query genome sequence. This was implemented on the website sequence query page (https://bigsdb.readthedocs.io/en/{PI}latest/administration.html#scheme-profile-clustering-setting-up-classification-schemes). This functionality returns the classification identifiers (including MLST-inherited CG and SL identifiers) of the cgMLST profile that is most closely related to the query genomic sequence, along with its number of mismatches compared with the closest profile.

cgLIN code functionality was also incorporated into BIGSdb version v1.34.0 (https://bigsdb.readthedocs.io/en/latest/administration.html#setting-up-lincode-definitions-for-cgmlst-schemes). In particular, cgST profiles can be queried by full cgLIN code or any prefix, and a nomenclature can be attached to LINcode prefixes of interest (e.g., SL258 is attached to prefix 0_0_1 and CG258 to 0_0_105_6).

Note that the identification of users’ query genomic sequences is made possible either through the BIGSdb platform that underlies the cgMLST website or externally after the export of the cgMLST profiles, which are publicly accessible.

## Discussion

The existence within microbial species of SLs with unique genotypic and phenotypic properties underlines the need for infraspecific nomenclatures (Maiden et al. 1998; Lan and Reeves 2001; Rambaut et al. 2020). Similar to species and higher Linnaean taxonomic ranks, a strain taxonomy should: (1) recognize genetic discontinuities and capture the most relevant SLs at different phylogenetic depths; (2) provide an unambiguous naming system for SLs; and (3) provide identification methods for placement within the taxonomic framework. Here, we developed a strain taxonomy consisting of a dual naming system that is grounded in population genetics and linked to an identification tool. The proposed system thus complies with the three fundamental pillars of taxonomy.

Although 7-gene MLST has been widely adopted as a taxonomic system of KpSC strains, several limitations are apparent: besides its restricted resolution, MLST identifiers do not convey phylogenetic information, as a single nucleotide substitution generates a different ST with unapparent relationships with its ancestor. Further, approximately half of the ST partitions were not monophyletic. The cgMLST approach provides much higher resolution and phylogenetic precision (Maiden et al. 2013; Zhou et al. 2021). Although other metrics such as whole-genome SNPs or ANI can be used to classify strains (Marakeby et al. 2014; Dallman et al. 2018), cgMLST presents advantages inherited from classical MLST, including standardization, reproducibility, portability and the conversion of sequences into human-readable allelic numbers. The high reproducibility and easy interpretation of cgMLST are two critical characteristics for its adoption in epidemiological surveillance. Here, we showed that cgMLST, based on 629 genes, has a much broader dynamic range than ANI when considering intraspecific variation (supplementary fig. S4, Supplementary Material online), and enables defining several hierarchical classification levels (Zhou et al. 2021). The resolutive power of the 629-loci cgMLST scheme provides valuable genotyping discrimination up to outbreak resolution and is highly consistent with whole-genome SNPs (Miro et al. 2020). However, to define shallower genetic structure within SLs resulting from recent clonal expansions or outbreaks, higher resolution should be sought based, for example, on core gene sets of specific SLs.

Optimization of threshold definitions based on population structure aims at optimizing cluster stability (Barker et al. 2018; Zhou et al. 2021). The density distribution of pairwise allelic mismatch dissimilarities within *K. pneumoniae* and related species exhibited genetic discontinuities at several phylogenetic depths. We took the benefit of this multimodal distribution to define optimal intraspecific classification thresholds and combined a clustering consistency coefficient (Silhouette) with a newly developed strategy that evaluates cluster stability by subsampling the entire data set. We defined four classifications at phylogenetic depths that reflected natural discontinuities within the population structure of *K. pneumoniae*, including the deep subdivisions of *K. pneumoniae sensu lato*. The 190-mismatch SL level was designed to capture the numerous deep phylogenetic branches within phylogroups (Bialek-Davenet et al. 2014; Holt et al. 2015). In turn, the CG level was useful to capture the genetic structuration observed within these primary SLs. For example, the CG-level nomenclature captures the evolutionary split of CG258 and CG11 from their SL258 ancestor, caused by a 1.1 Mb recombination event (Chen et al. 2014) (supplementary fig. S5, Supplementary Material online).

Seven-locus MLST is a widely adopted nomenclature system, as illustrated by the widespread use of ST identifiers associated with hypervirulent or multidrug-resistant SLs (e.g., “*Klebsiella pneumoniae* ST258”: 293 PubMed hits; ST23: 117 hits; on July 20, 2021). Backward nomenclatural compatibility is therefore critical. After applying our inheritance algorithm, most SLs and CGs were labeled according to the 7-gene MLST identifier of the majority of their isolates. Widely adopted ST identifiers will therefore designate nearly the same strain groups within the proposed genomic taxonomy of *K. pneumoniae*, which should greatly facilitate its adoption. We note that the 7-gene MLST nomenclature will still have to be expanded, as this classical approach continues to be widely used. However, for practical reasons, upcoming MLST and cgMLST nomenclatural identifiers will be uncoupled, and we suggest that the cgMLST-based identifiers of SLs and CGs (rather than their ST) should be adopted as the reference nomenclature in the future.

Instability is a major limitation of single linkage clustering, caused by group fusion known as the chaining effect (Turner and Feil 2007). This is particularly relevant over epidemiological timescales, where intermediate genotypes (e.g., a recent ancestor or recombinant) are often sampled (Feil 2004). This issue is exacerbated in *K. pneumoniae*, where large-scale recombination may result in truly intermediate genotypes (Chen et al. 2014; Holt et al. 2015), referred to as “hybrids” by analogy to eukaryotic biology. Here, this phenomenon was illustrated through our delayed introduction into our nomenclature, of 138 interphylogroup hybrid genomes. The merging of predefined classification groups can be handled by classification versioning or ad hoc rules (Zhou et al. 2021), but this is indeterministic and challenging in practice.

To address its stability issue, we complemented the single linkage clustering approach with a fully stable approach. LIN codes were proposed as a universal genome coding system (Marakeby et al. 2014; Tian et al. 2020), a key feature of which is the generation of definitive genome codes that are inherently stable. The original LIN code system was based on the ANI metric; here, we noted that the ANI values that best correspond to some of the 10 cgMLST thresholds were highly similar (supplementary table S8, Supplementary Material online), casting doubt on the reliability of this metric for small-scale genetic distances. In addition, the ANI metric is nonreciprocal and highly dependent on comparison implementations and parameters. These shortfalls may yield imprecision and nonreproducibility that are particularly impactful for comparisons between very similar genomes. We, therefore, adapted the LIN code concept to cgMLST-based similarity (i.e., one-complement of the allelic mismatch proportions) to classify strains into a cgLIN code system. This strategy leverages the benefits of cgMLST and introduces more intuitive shallow-level classification thresholds. The multilevel similarity information embedded in MLSL and cgLIN “barcodes” provides a human-readable snapshot of strain relationships, as nearly identical genomes have identical barcodes up to a position near the right end. In contrast to single linkage clustering partitions, one important limitation of LIN codes is that preexisting classification identifiers (e.g., ST258) cannot be mapped onto individual LIN code identifiers, because these are attributed with reference to the upper levels and are set to 0 for each downstream level (Marakeby et al. 2014). However, cgLIN code prefixes may represent useful labels for particular lineages.

The cgMLST-based nomenclatures have some limitations. First, a comparison of allele numbers rather than SNPs implies the loss of information. In turn, this approach is advantageous to estimate evolutionary relationships of closely related genomes that diverged following homologous recombination events, which are common among strains within bacterial species (Feil 2004; Vos and Didelot 2009). Second, MLST-based distances saturate more rapidly than SNP distances (once a locus is affected, even by a single mutation, further mutations at this locus will change the allele but will not increase the allelic distance), and are therefore mostly meaningful within bacterial species. Third, in contrast to the original ANI-based LIN code approach (Tian et al. 2020), cgMLST-based LIN codes require prior development of cgMLST schemes, which are larger, hence more powerful for strain resolution, for single species. Therefore, the advantages of cgLIN codes for population genomics and epidemiological questions come at the expense of universality. Still, the dual MLSL and cgLIN code approach proposed here is in principle applicable to all bacterial species (or closely related groups thereof) for which large representative sets of genomes are available. The cgLIN code algorithms were incorporated into BIGSdb and should be readily portable to other existing cgMLST platforms such as EnteroBase. However, the use of genetic thresholds for an entire group of organisms may not always be meaningful, depending on the population genetic structure. Whereas *K. pneumoniae* shows strong structuring with neat peaks and valleys of pairwise genetic distances, other species may have a more fuzzy structure. In the latter cases, the approach will still be applicable, but even optimally defined thresholds may be less relevant biologically.

### Conclusions

A unified nomenclature of pathogen genotypes is required to facilitate communication in the “One Health” and “Global Health” perspectives. *K. pneumoniae* represents a rapidly growing public health threat, and the availability of a common language to designate its emerging SLs is therefore highly timely. The proposed unified taxonomy of *K. pneumoniae* strains will facilitate advances on the biology of its SLs across niches, time, and space, and will endow surveillance networks with the capacity to efficiently monitor and control the emergence of SLs of high public health relevance.

Here, we propose a dual barcoding approach to bacterial strain taxonomy, which combines the complementary advantages of stability provided by the cgLIN codes, with an unstable, but human-readable MLSL nomenclature rooted in the popular 7-gene MLST nomenclature. Because they are definitive, cgLIN codes can be used for the traceability of cluster fusions that will occur occasionally in the MLSL arm of the dual taxonomy (supplementary fig. S11, Supplementary Material online). We contend the stability of cgLIN codes and their use alongside MLSL approaches provide a pragmatic solution to current attempts at developing genomic taxonomies of bacterial strains that are both stable and practical for human-to-human communication.

## Materials and Methods

### Definition of an Updated Core Genome MLST (scgMLSTv2) Genotyping Scheme

We previously defined a cgMLST (using *strict* synteny criteria, hence name scgMLSTv1) scheme of 634 highly syntenic genes (Bialek-Davenet et al. 2014). Here, we updated the scgMLSTv1 scheme, with the following improvements. First, two loci (KP1_2104 and *aceB* = KP1_0253) were removed because they were absent or truncated in multiple strains, based on 751 high-quality assemblies available in the BIGSdb-Pasteur *Klebsiella* database on October 16, 2017 (project id 11 at https://bigsdb.pasteur.fr/cgi–bin/bigsdb/bigsdb.pl?db=pubmlst_klebsiella_isolates). Second, the remaining 632 loci templates were modified so that they would include the start and stop codons of the corresponding coding sequence (CDS). This was not the case for all CDSs of the scgMLSTv1 scheme, as some loci corresponded to internal portions of CDSs. These template redefinitions were done to harmonize locus definitions across the scheme. Of note, defining loci as complete CDSs also facilitates genotyping, by enabling precise identification of the extremities of novel alleles, through the search of the corresponding start and stop codons. As a result of these locus template extensions, three additional loci (yraR, rnt and KP1_1655) had to be removed because they were called in a low proportion of the above 751 genomes. The resulting 629 scgMLSTv2 genes have a summed length of 512,856 nt (9.8% of the genome of reference strain NTUH-K2044), when compared with 507,512 nt (9.7%) for the corresponding loci in scgMLSTv1.

### Definition of a Genomic Sequence Data set of 7,060 Isolates with cgMLST Profiles

The KpSC comprises seven phylogroups that have been given taxonomic status in the prokaryotic nomenclature: *K. pneumoniae* subsp. *pneumoniae* (Kp1, also known as *K. pneumoniae sensu stricto*), *K. quasipneumoniae* subsp. *quasipneumoniae* (Kp2), *K. variicola* subsp. *variicola* (Kp3), *K. quasipneumoniae* subsp. *similipneumoniae* (Kp4), *K. variicola* subsp. *tropica* (Kp5), “*K. quasivariicola*” (Kp6) and *K. africana* (Kp7) (Rodrigues et al. 2019). We retrieved all KpSC genomes from the GenBank assembly repository on March 15, 2019, corresponding to 8,125 assemblies. We then chose high-quality assemblies by excluding draft genomes: (1) containing more than 1,000 contigs of size >200 nt; (2) for which the ANI values (estimated using FastANI v1.1) were <96% against every reference strain of the taxonomic diversity of the SC (Rodrigues et al. 2019; supplementary table S9, Supplementary Material online); (3) of size ≤ 4.5 or ≥ 6.5 Mb; and (4) with G + C% content >59%. The data of each criterion per strain are shown in supplementary table S1, Supplementary Material online. The three last criteria excluded possible contamination or non-KpSC genomes (supplementary fig. S2, Supplementary Material online).

The resultant 7,388 “high-quality” draft genomes (supplementary table S2, Supplementary Material online) were scanned for scgMLSTv2 alleles, using the BLASTN algorithm, as implemented in the BIGSdb platform (Jolley and Maiden 2010; Jolley et al. 2018), with 90% identity, 90% length coverage, word size 30, with type alleles only (as defined below). After this step, 235 profiles were excluded because they had more than 30 missing alleles.

The resulting data set comprised 36 taxonomic references of Kp1–Kp7 (Rodrigues et al. 2019) that, together with eight additional genomes of phylogroup Kp7, were considered as a reference data set of the KpSC taxa (“*K. quasivariicola*” reference strain KPN1705, SB6096, was excluded because it had more than 30 missing alleles). Besides these 44 reference genomes, 7,154 GenBank genomes were retained, resulting in a total data set of 7,198 genomes (supplementary tables S1 and S2, Supplementary Material online). For some analyses, 138 genomes were set aside, defined as “hybrids” between phylogroups (see below), resulting in a 7,060-genome data set (supplementary fig. S2, Supplementary Material online).

We estimated within-outbreak variation using previously published outbreak sets (supplementary tables S6 and S7, Supplementary Material online).

### Recording Sequence Variation at the cgMLST Gene Loci

Allelic variation at scgMLSTv2 loci was determined with the following strategy. First, the sequence of strain NTUH-K2044 was used as the reference genome, with all its alleles defined as allele 1. Then, BLASTN searches (70% identity, 90% length coverage) were carried out using allele 1 as query against the genomic sequences of reference genomes 18A069, 342, 01A065, 07A044, CDC4241-71, and 08A119, representing major lineages (phylogroups Kp2–Kp6, including two genomes of Kp3 and excluding Kp7, which was not discovered yet) of the KpSC (Blin et al. 2017). Only sequences with a complete CDS (start and stop, no internal frameshift) and within a ±5% range of the reference size were accepted. Alleles defined from these reference genomes and from NTUH-K2044 were then defined as type alleles.

New alleles were identified by BLASTN searches using a 90% identity threshold, 90% length coverage, and a word size of 30 and the above-defined type alleles. The use of type alleles avoided expanding the sequence space of alleles in an uncontrolled way, at the cost of losing a few highly divergent alleles, which may have replaced original (vertically inherited) alleles by HGT and homologous recombination. As for type alleles, novel alleles were accepted only if they (1) corresponded to a complete CDS (start and stop codons with no internal frameshift mutations) and (2) were within a 5% (plus/minus) of the size of the type allele size. Novel allele sequences were also excluded if they came from assemblies with more than 500 contigs of size >200 nt, as these may correspond to low-quality assemblies and that might contain artifactual alleles. Genome assemblies based on 454 sequencing technology, which are prone to frameshifts, were also excluded for novel allele definitions. No genome assemblies based on IonTorrent sequencing technology were found.

In order to speed the scanning process, we used the fast scan option (-e -f) of the BIGSdb autotag.pl script (https://bigsdb.readthedocs.io/en/latest/offline_tools.html). This option limits the BLASTN search to a few exemplar alleles, which are used as query to find the genomic region corresponding to the locus. In a second step, a direct database lookup of the region was performed to identify the exact allele.

### Definition of MLST STs and cgST

Classical 7-gene MLST loci have been defined previously (Diancourt et al. 2005) as internal portions of the seven protein-coding genes *gapA*, *infB*, *mdh*, *pgi*, *phoE*, *rpoB*, and *tonB*. Novel alleles were defined in the Institut Pasteur *Klebsiella* MLST and whole-genome MLST database https://bigsdb.pasteur.fr/klebsiella. In 7-locus MLST, the combination of the seven allelic numbers determines the isolate profile, and each unique profile is attributed an ST number. Incomplete MLST profiles with one (or more) missing gene(s) are recorded in the isolates database, but in these cases, no ST number can be attributed and the profiles are therefore not defined in the sequence definition database. The 7-locus MLST genes were not included in the scgMLSTv2 scheme.

Similar to ST identifiers used for unique 7-gene MLST allelic combinations, each distinct cgMLST profile can be assigned a unique identifier; however, when using draft genomes, cgMLST data can be partly incomplete due to de novo assembly shortcomings or missing loci. cgSTs were therefore defined only for cgMLST profiles with no more than 30 uncalled alleles out of the 629 cgMLSTv2 loci. In addition, we have used the –match_missing option of the define_profiles.pl script, which allows missing loci to be treated as specific alleles rather than “any” alleles. While this retains more information (because it differentiates profiles that differ only by missing data at different loci), it can result in some isolates genomes corresponding potentially to more than a single cgST; their equality can nevertheless be deduced by the last MLSL level, “mismatch 0,” as these will be grouped into the same clusters at level 0; this is because the clustering ignores loci with missing data in any of the profiles of a pair when calculating the pairwise distance. For example, cgST1 = 0–N–1–1; cgST2 = 0–2–N–1; an isolate with profile 0–2–1–1 would result in cgST3 = 0–2–1–1 being created, and this genome would equate to both previous cgSTs and would be labeled as cgST1; cgST2; cgST3.

### Phylogenetic Analyses, Recombination Tests, and Screens for Virulence and Resistance Genes

JolyTree v2.0 (Criscuolo 2019, 2020) was used to reconstruct a phylogenetic tree of the KpSC. For this, first, a single linkage clustering was performed to cluster cgSTs into partitions. This clustering was applied on the pairwise distances between allelic profiles, defined as the number of loci with different alleles, normalized by the number of loci with alleles called in both profiles. A threshold of 8 mismatches was defined, resulting in 2,417 clusters. One genome from each of these 2,417 clusters was used as an exemplar for phylogenetic analysis (fig. 1).

A core genome multiple sequence alignment (cg-MSA) of 7,060 cgMLST profiles free of evidence for interphylogroup “hybridization” (see below) was constructed. The gene sequences were retrieved based on allele number in the sequence definition database, individual gene sequences were aligned with MAFFT v7.467 (missing alleles were converted into gaps), and the multiple sequence alignments were concatenated. IQ-TREE v2.0.6 was used to infer a phylogenetic tree with the GTR + G model (fig. 2).

Locus-by-locus recombination analyses were computed with the PHI test (Bruen et al. 2006) using PhiPack v1.0.

Kleborate v2.0.4 (Lam et al. 2021) was employed to identify acquired antimicrobial resistance and virulence genes in genomic sequences, based on CARD v3.0.8 database, with identity >80% and coverage >90%. Virulence score (ranges from 0 to 5) and antimicrobial resistance score (ranges from 0 to 3) were also derived from Kleborate. The virulence score is assigned according to the presence of yersiniabactin (*ybt*), colibactin (*clb*), and aerobactin (*iuc*), as follows: 0 = none present, 1 = yersiniabactin only, 2 = yersiniabactin and colibactin (or colibactin only), 3 = aerobactin (without yersiniabactin or colibactin), 4 = aerobactin and yersiniabactin (without colibactin), and 5 = all three present. Resistance scores are calculated as follows: 0 = no ESBL (Extended-Spectrum Beta-Lactamases), no carbapenemase, 1 = ESBL without carbapenemase, 2 = carbapenemase without colistin resistance, 3 = carbapenemase with colistin resistance.

### Detection of Hybrid Genomes

HGT of large portions of the genome can occur among isolates belonging to distinct KpSC phylogroups (Holt et al. 2015). Additionally, MLST or scgMLST alleles may have been transferred horizontally from non-KpSC members, for example, *Escherichia coli*. For the purpose of phylogeny-based classification, putative hybrid genomes were excluded. To define genomes that result from large interphylogroup recombination events, the gene-by-gene approach was used to define an original strategy, outlined briefly here and more thoroughly in supplementary appendix (Detection of hybrids), Supplementary material online: for each locus, each allele was unambiguously labeled by one of the seven KpSC phylogroup of origin, if possible; next, for each profile, a phylogroup homogeneity index (i.e., proportion of alleles labeled by the predominant phylogroup) was derived. The distributions of the phylogroup homogeneity indices allowed determining hybrid genomes (supplementary fig. S12, Supplementary Material online). The exclusion of such hybrid genomes resulted in a genomic data set of 7,060 isolates deemed as having a majority of alleles inherited from within a single phylogroup. Of the 44 reference genomes, one (SB1124, of phylogroup Kp2) was defined as having a hybrid origin: 414 alleles were attributed to Kp2, whereas 150 alleles originated from non-KpSC species; as a result, 73.4% of SB1124 alleles were part of the majority phylogroup, which was below the defined threshold of 78%. The quantification of recombination breakpoints was performed based on the position of cgMLST loci on the NTUH-K2044 reference genome (NC_012731), counting the number of recombination breakpoints in each successive 500 kb fragment along the reference genome. Note that hybrids had typical assembly sizes (supplementary table S1, Supplementary Material online), whereas our simulations of contaminated sequence read sets between phylogroups resulted in significantly larger assemblies (not shown; available upon request).

### Identification of Genetic Discontinuities in the KpSC Population Structure

The tool MSTclust v0.21b (https://gitlab.pasteur.fr/GIPhy/MSTclust) was used to perform the single linkage clustering of cgMLST profiles from their pairwise allelic mismatch dissimilarities, as well as to assess the efficiency of the resulting profile partitioning (for details, see supplementary appendix: Minimum Spanning tree-based clustering of cgMLST profiles, Supplementary material). Briefly, for each threshold *t* (= 0–629 allelic mismatches), the clustering consistency was assessed using the silhouette metrics *S_t_* (Rousseeuw 1987), whereas its robustness to profile subsampling biases was assessed using a dedicated metrics *W_t_* based on the adjusted Wallace coefficients (Wallace 1983; Severiano et al. 2011). Both consistency (*S_t_*) and stability (*W_t_*) coefficients converge to 1 when the threshold *t* leads to a clustering that is consistent with the “natural” grouping and is robust to subsampling biases, respectively.

The adjusted Rand index *R_t_* (Hubert and Arabie 1985; Carrico et al. 2006) was used to assess the global concordance between single linkage clustering partitions and those induced by classifications into 7-gene MLST STs, subspecies, and species.

### Diversity and Phylogenetic Compatibility Indices

Simpson’s diversity index was computed using the www.comparingpartitions.info website (Carrico et al. 2006). The clade compatibility index of STs or other groups was calculated using the ETE Python library (http://etetoolkit.org/{PI}docs/latest/tutorial/tutorial_trees.html#checking-the-monophyly-of-attributes-within-a-tree), in order to define whether their constitutive genomes formed a monophyletic, paraphyletic, or polyphyletic group within the recombination-purged sequence-based phylogeny of the core genome. We estimated clade compatibility as the proportion of nonsingleton STs, SLs, or CGs that were monophyletic.

### Classification of cgMLST Profiles into Clonal Groups and SLs

The classification scheme functionality was implemented within BIGSdb v1.14.0 and relies on single linkage clustering. Briefly, cgSTs were defined in the sequence definitions (“seqdef”) database as distinct profiles with fewer than 30 missing alleles over the scgMLST scheme, and their pairwise cgMLST distance was computed as the number of distinct alleles. To account for missing data, a relative threshold was used for clustering: the number of allelic mismatches was multiplied by the proportion of loci for which an allele was called in both strains. Hence, in order to be grouped, the number of matching alleles must exceed: (the number of loci called in both strains × (total loci − defined threshold))/total loci. cgSTs and their corresponding SL, CG, and other levels partition identifiers are stored in the seqdef database and are publicly available. Here, classification schemes were defined in the *Klebsiella* seqdef database on the top of the scgMLSTv2 scheme, and host single linkage clustering group identifiers at the 10 defined cgMLST allelic mismatch thresholds (see Results). For classification groups defined using 43 and 190 allelic mismatch thresholds, scheme fields were defined and populated with the identifiers defined by inheritance from 7-gene MLST ST identifiers (see supplementary appendix: Nomenclature inheritance algorithm, Supplementary material online).

### Adaptation of the LIN Code Approach to cgMLST: Defining cgLIN Codes

Vinatzer and colleagues proposed an original nomenclature method in which each genome is attributed a LIN code, based on genetic similarity with the closest previously encoded member of the nomenclature (Marakeby et al. 2014; Weisberg et al. 2015; Vinatzer et al. 2016, 2017; Tian et al. 2020). In this proposal, the similarity between genomes was based on ANI ((Konstantinidis and Tiedje 2005; Goris et al. 2007)), with a set of 24 thresholds corresponding to ANI percentages of 60, 70, 75, 80, 85, 90, 95, 98, 98.5, 99, 99.25, 99.5, 99.75, 99.9, 99.925, 99.95, 99.975, 99.99, 99.999, and 99.9999. Here, the method was adapted by replacing the ANI metric by the similarity between cgMLST profiles, defined as the proportion of loci with identical alleles normalized by the number of loci with alleles called in both profiles. These codes, which we refer to as cgLIN codes, are composed of a set of *p* positions, each corresponding to a pairwise genome similarity threshold *s_p_*. These similarity thresholds are sorted in ascending order (i.e., *s_p_* < *s_p_*_+1_), the first positions of the code (on the left side) thus corresponding to low levels of similarity. Following the initial proposal, the codes are assigned as follows (supplementary fig. S13, Supplementary Material online): (step 1) the code is initialized with the first strain being assigned the value “0” at all positions; (step 2) the encoding rule for a new genome *i* is based on the closest genome *j* already encoded as follows, from the similarity *s_ij_* ∊ ] *s_p–_*_1_, *s_p_*]:

- identical to code *j* up to and including position *p* − 1;
- for the position *p*: maximum value observed at this position (among the subset of codes sharing the same prefix at the position *p* − 1) incremented by 1;
- “0” to all downstream positions, from *p* + 1 included.

For each genome to be encoded, step 2 is repeated.

A set of 10 cgMLST thresholds were defined as follows: first, four thresholds were chosen above the similarity values peak observed between *Klebsiella* species (*s_p_* = 1–610/629 = 0.03), subspecies (*s_p_* = 1–585/629 = 0.07), main SLs (*s_p_* = 1–190/629 = 0.70), and CGs (*s_p_* = 1–43/629 = 0.93). Second, we included six thresholds deemed useful for epidemiological studies, corresponding to 10, 7, 4, 2, 1, and 0 allelic mismatches.

This encoding system conveys phylogenetic information, as two genomes with identical prefixes in their respective cgLIN codes can be understood as being similar, to an extent determined by the length of their common prefix. Isolates having cgMLST profiles with 100% identity (no mismatch at loci called in both genomes) will have exactly the same cgLIN code. For example, cgLIN codes 0_0_22_12_0_1_0_0_0_0 and 4_0_3_0_0_0_0_0_0_0 would denote two strains belonging to distinct species (as they differ by their first number in the code). cgLIN codes 0_0_105_6_0_0_75_1_1_0 and 0_0_105_6_0_0_75_1_0_0 correspond to strains from Kp1 (prefix 0_0) that differ by only 2 loci; they are identical up to the second bin, corresponding to 2 locus mismatches (supplementary fig. S13, Supplementary Material online); note that 0 and 1 mismatches are both included in the last bin: genomes have an identical identifier when having 0 difference, and a different identifier when having 1 mismatch (supplementary fig. S14, Supplementary Material online).

The impact of genome input order on the number of cgLIN code partitions at a given threshold was defined using the 7,060 high-quality, nonhybrid cgMLST profiles, which were encoded 500 times with random input orders (see details in the supplementary appendix: Impact of strains input order on LIN codes, and use of Prim’s algorithm, Supplementary material online).

The scripts for cgLIN code database creation were made available via GitLab BEBP (https://gitlab.pasteur.fr/BEBP/LINcoding).

## Supplementary Material

Supplementary data are available at *Molecular Biology and Evolution* online.

## Supplementary Material

msac135_Supplementary_Data

## Data Availability

All genome assembly accessions are provided in Supplementary Tables S1 and S9. The allele sequences for each of the 629 loci of the scgMLSTv2 scheme are available at https://bigsdb.pasteur.fr/cgi-bin/bigsdb/bigsdb.pl?db=pubmlst_klebsiella_seqdef&page=downloadAlleles&scheme_id=18. The source codes of MSTclust and LINcoding are available at https://gitlab.pasteur.fr/GIPhy/MSTclust and https://gitlab.pasteur.fr/BEBP/LINcoding, respectively.
